# Repeated exposure of the oral mucosa over 12 months with cold plasma is not carcinogenic in mice

**DOI:** 10.1038/s41598-021-99924-3

**Published:** 2021-10-19

**Authors:** K. Evert, T. Kocher, A. Schindler, M. Müller, K. Müller, C. Pink, B. Holtfreter, A. Schmidt, F. Dombrowski, A. Schubert, T. von Woedtke, S. Rupf, D. F. Calvisi, S. Bekeschus, L. Jablonowski

**Affiliations:** 1 Institute of Pathology, University of Regensburg, Franz-Josef-Strauß-Allee 11, 93053 Regensburg, Germany; 2 Department of Restorative Dentistry, Periodontology, Endodontology, and Preventive and Pediatric Dentistry, University Medicine Greifswald, Greifswald, Germany; 3 Leibniz Institute of Surface Modification (IOM Leipzig), Leipzig, Germany; 4 Consultants PILOTO, Ion Beam & Plasma Surface Technologies, Grimma, Germany; 5 Center for Clinical Studies, University Hospital Regensburg, Regensburg, Germany; 6 ZIK Plasmatis, Leibniz Institute for Plasma Science and Technology (INP), Greifswald, Germany; 7 Institute of Pathology, University Medicine Greifswald, Greifswald, Germany; 8 Department of Immunology, Fraunhofer Institute for Cell Therapy and Immunology (IZI), Leipzig, Germany; 9 Department of Hygiene and Environmental Medicine, University Medicine Greifswald, Greifswald, Germany; 10 Clinic of Operative Dentistry, Saarland University, Homburg, Germany

**Keywords:** Dentistry, Therapeutics, Cancer

## Abstract

Peri-implantitis may result in the loss of dental implants. Cold atmospheric pressure plasma (CAP) was suggested to promote re-osseointegration, decrease antimicrobial burden, and support wound healing. However, the long-term risk assessment of CAP treatment in the oral cavity has not been addressed. Treatment with two different CAP devices was compared against UV radiation, carcinogen administration, and untreated conditions over 12 months. Histological analysis of 406 animals revealed that repeated CAP exposure did not foster non-invasive lesions or squamous cell carcinoma (SCCs). Carcinogen administration promoted non-invasive lesions and SCCs. Molecular analysis by a qPCR screening of 144 transcripts revealed distinct inflammatory profiles associated with each treatment regimen. Interestingly, CAP treatment of carcinogen-challenged mucosa did not promote but instead left unchanged or reduced the proportion of non-invasive lesions and SCC formation. In conclusion, repeated CAP exposure of murine oral mucosa was well tolerated, and carcinogenic effects did not occur, motivating CAP applications in patients for dental and implant treatments in the future.

## Introduction

Dental implant installment is a widespread and accepted treatment method to replace missing teeth. Implantation, however, frequently spurs peri-implantitis, which may lead to implant loss. In a population-based Swedish study, 15% of all patients (8% of implants) had peri-implantitis at a 9-year follow-up^1^. The removal of biofilm from the exposed implant surface is regarded as the gold standard peri-implantitis therapy. Various implant surface decontamination methods have been used, but a meta-analysis of decontamination studies did not find any technique showing superior clinical outcomes, and no approach achieved long-lasting results^2^. This is mainly because current methods lack fully efficient removal of bacterial biofilms, allowing residual microorganisms to re-populate the surface and promote inflammation and peri-implantitis^3^. Thus, there is a need to develop new tools for successful peri-implantitis treatment.

Recently, cold atmospheric pressure plasma (CAP) was considered a possible and innovative treatment option to bridge this therapeutic gap^4^. Physical plasmas are the so-called "fourth state of matter." These partially ionized, energetic gases are electrically neutral and composed of ions, electrons, low-level ultraviolet (UV) radiation, moderate thermal radiation, and reactive oxygen and nitrogen species (ROS/RNS)^5^. These species' short lifespan is a desirable effect, as the CAP treatment does not leave any residuals on the target tissue as observed with, for instance, pharmaceutical compounds. CAP inactivates planktonic bacteria, yeast, and spores^6^ and hydrophilizes target surfaces^7^, while the extent of these effects depends on CAP intensity and treatment time^8^. CAP devices designed for medical purposes deliver therapeutic ROS/RNS while being operated at body temperature (not exceeding 40 °C), making them suitable to be applied to heat-sensitive surfaces and human tissue^9^.

CAP has different active components such as UV radiation, free radicals, ozone, and electrical charges. At larger concentrations or intensities, some of these components are known drivers of carcinogenesis. Therefore, CAP effects on the oral mucosa must be evaluated carefully to unravel any potential side effects before application in humans.

Previously, we treated the cheek mucosa of mice with two different CAP devices or UV radiation for up to 1 min and performed histological analysis against untreated controls after 1 day and a week^10^. Focal mucosal ulceration and necrosis accompanied by a mild inflammatory reaction observed on day one had been entirely re-epithelialized at day seven. Thus, it is informative to study the consequences of a single CAP application in tissues, while in clinical applications, however, a repeated treatment regimen is needed.

Therefore, we investigated the long-term risk of repeated CAP applications to include potential carcinogenic effects that might be a consequence of the mild mucosal defects observed in the short-term study. Specifically, we designed a 1-year study in mice to investigate whether repeated CAP treatment either provokes preneoplastic lesions and squamous cell carcinoma or is well-tolerated in the long term. Two different CAP devices were tested in parallel against UV radiation and the co-carcinogen dibenzo(a)pyrene (DBP, the most carcinogenic component of tobacco smoke) as well as untreated animals as controls^11^. Additionally, due to a high prevalence of smokers in the population, tobacco's co-carcinogenic effect was investigated by combining DBP and CAP treatment.

## Results

**Figure 1:**
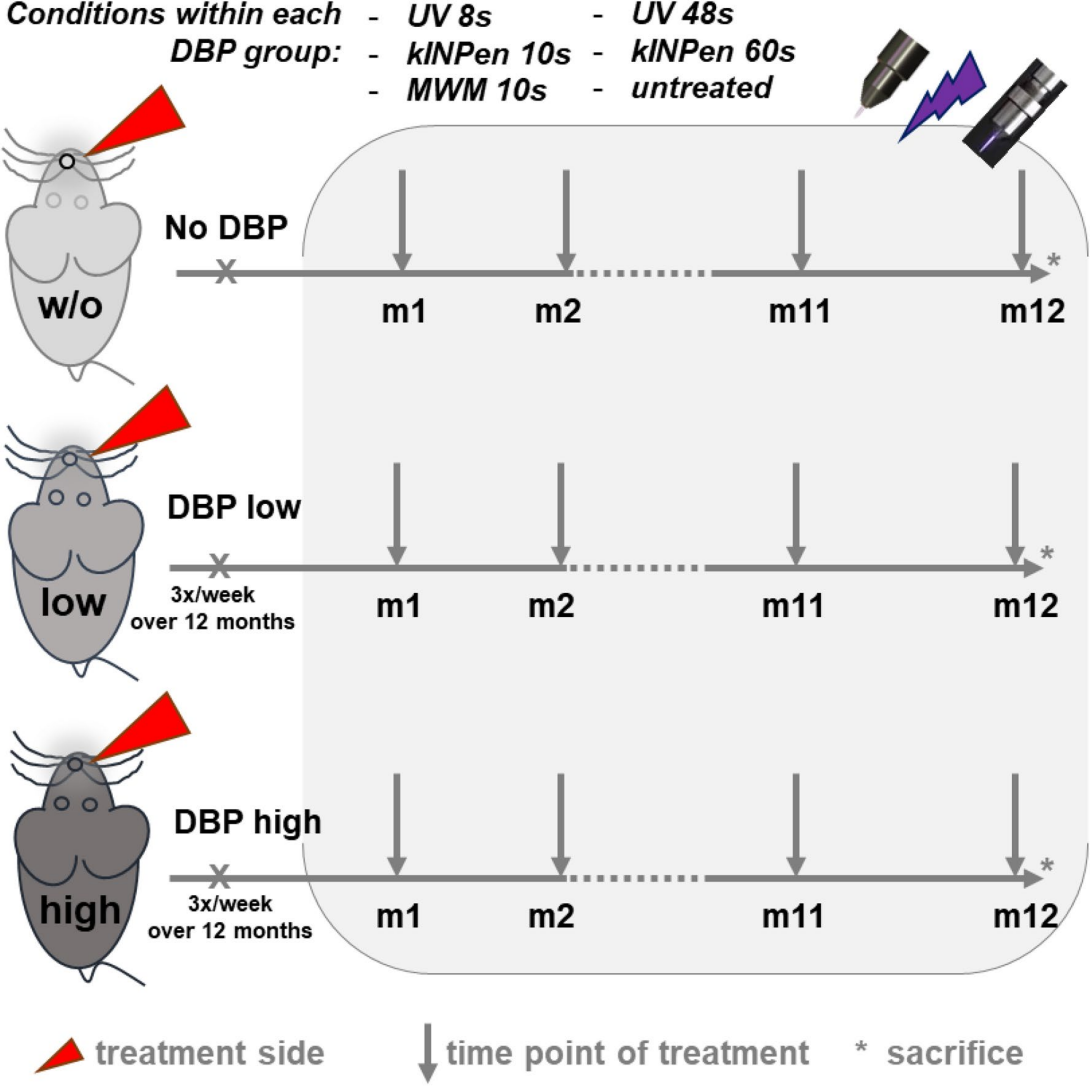
Flowchart of the study. Mice received either none or administrations of low (3 nmol) or high (24 nmol) concentrations of the co-carcinogenic substance dibenzo(a)pyrene (DBP) into the oral cavity three times per week over the entire study period of 12 months. The DBP administration was paralleled by a monthly treatment of the right cheek with either UV (ultraviolet) 8 s, UV 48 s, kINPen plasma 10 s, kINPen plasma 60 s, or MWM plasma 10 s across the period of 12 months. After sacrifice, sampling of tissue for downstream analyses was performed. Each group contained 19–26 animals.

We aimed to investigate the potential side effects of repeated cold atmospheric pressure plasma (CAP) treatment of the oral cavity in terms of tolerance, preneoplastic and squamous cell carcinoma (SCC) formation, preneoplastic and SCC size, and transcriptomic changes of the mucosa tissue (Fig. 1).

### CAP treatment does not harm the murine oral mucosa

As expected, across all groups, treatment with both the kINPen and MWM plasma source and UV were well tolerated. No treatment group showed increased mortality rates than the respective untreated animals within each of the DBP regimens (none, low dose DBP, high dose DBP). Animal scoring revealed no changes in behavior or grooming of the treatments within each regimen.

**Figure 2:**
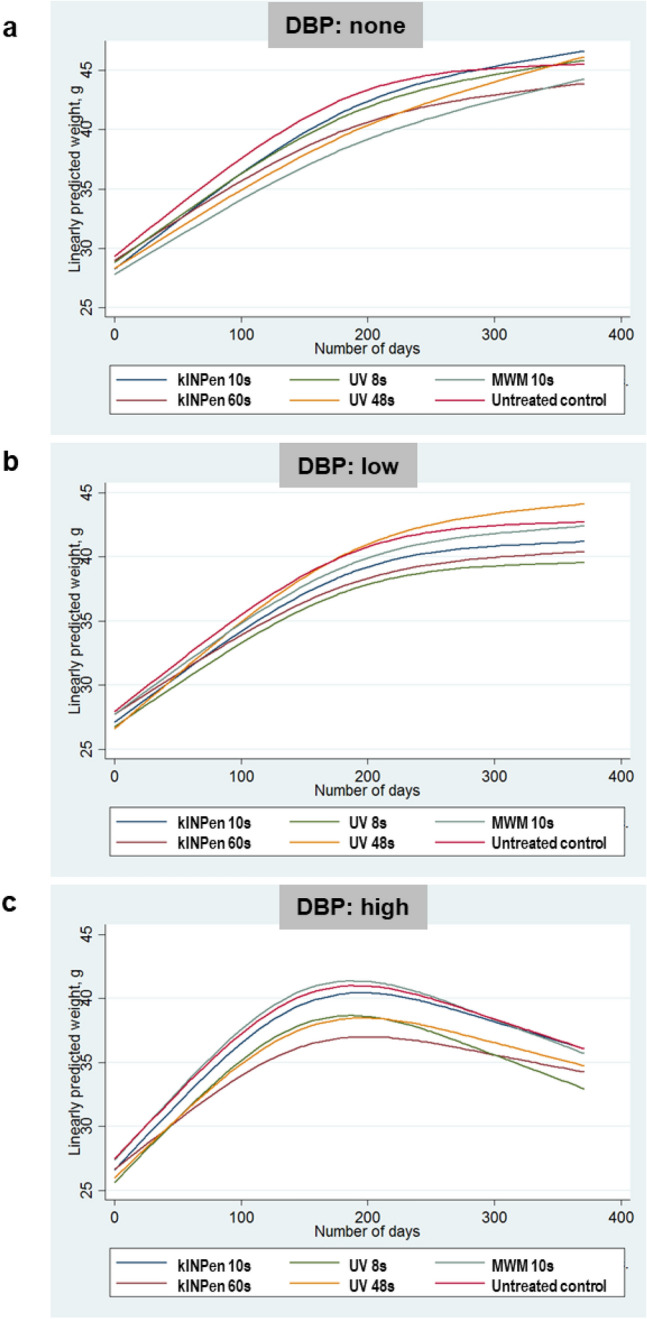
Animal weight gain. (**a**–**c**) Fitted animal weights across the 1-year observation period for the groups not receiving the co-carcinogenic substance dibenzo(a)pyrene (DBP) (**a**) as well as the groups receiving low (**b**) and high (**c**) doses of DBP.

For mice not receiving any DBP, weight increased from about 28 g to around 44–47 g after 1 year, and neither CAP nor UV treatment had a significant impact on the weight (Fig. 2a). In mice with low dose DBP, weight nearly plateaued after day 250 with a minimal upward change towards the end with final weights of 39–44 g (Fig. 2b). All mice with high dose DBP reached peak weights between day 180 and 200 at around 37–42 g. After that, they lost weight, and at sacrifice, they weighed between 33 and 36 g (Fig. 2c). In the DBP regimens, most treatment groups showed a statistically significant smaller weight increase over the entire observation period than controls.

### Repeated CAP treatment did not promote lesion or SCC formation

**Figure 3:**
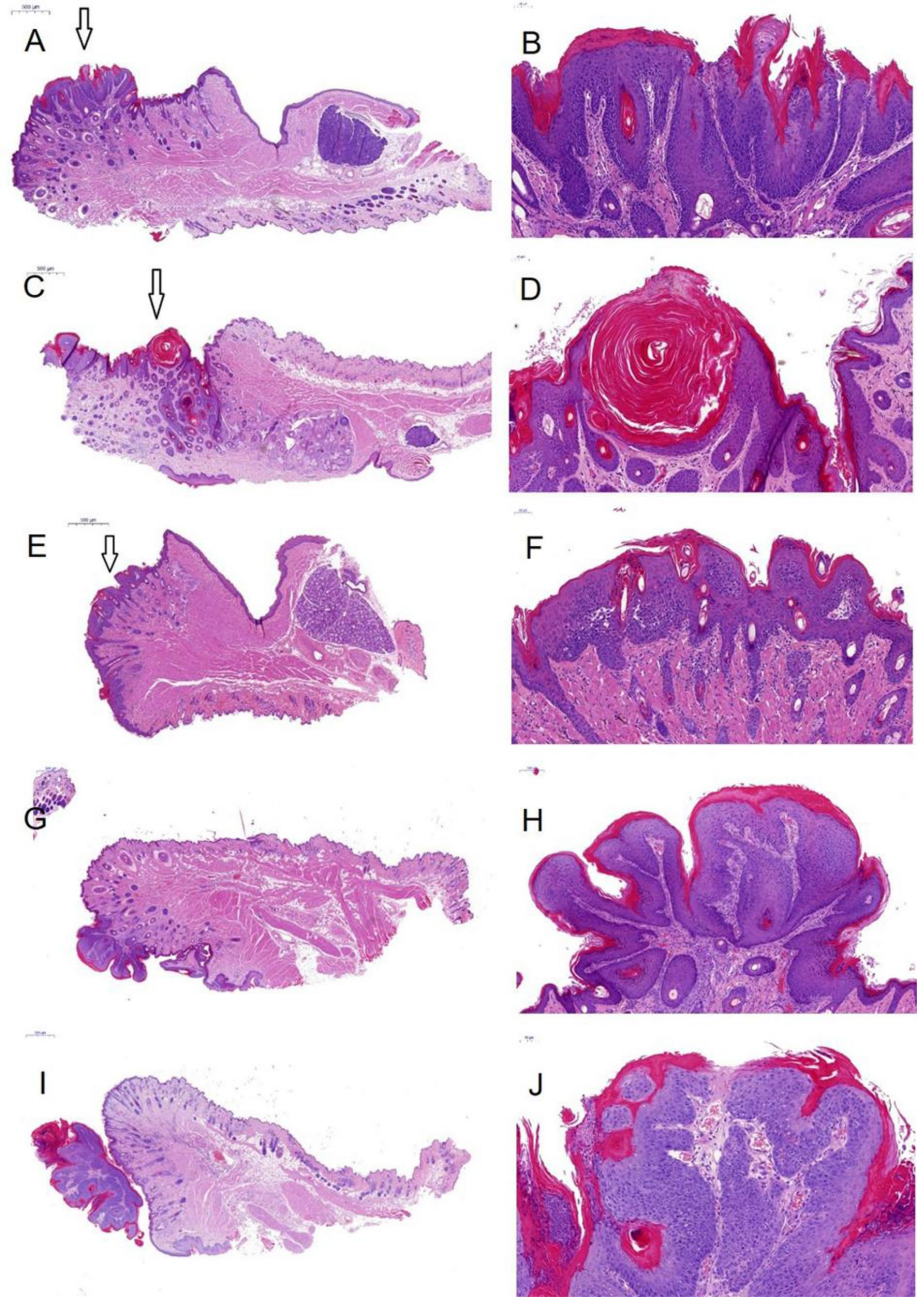
Representative histological analysis of non-invasive lesions of the mucosa. Haematoxylin–eosin staining of the oral mucosa of mice with different changes, summarized as non-invasive lesions [(**A**, **B**): hyperkeratosis with mild dysplastic changes; (**C**, **D**): hyperplasia and hyperkeratosis with mild to moderate dysplastic changes; (**E**, **F**): mild to moderate dysplastic changes; (**G**, **H**): papilloma without dysplasia; (**I**, **J**): papilloma with moderate dysplastic changes; (**A**, **B**): ultraviolet (UV) 8 s + dibenzo(a)pyrene (DBP) high dose; (**C**, **D**): UV 48 s + DBP high dose; (**E**, **F**): untreated + DBP high dose; (**G**, **H**): UV 8 s + DBP low dose; (**I**, **J**): kINPen 10 s + DBP low dose].

**Figure 4:**
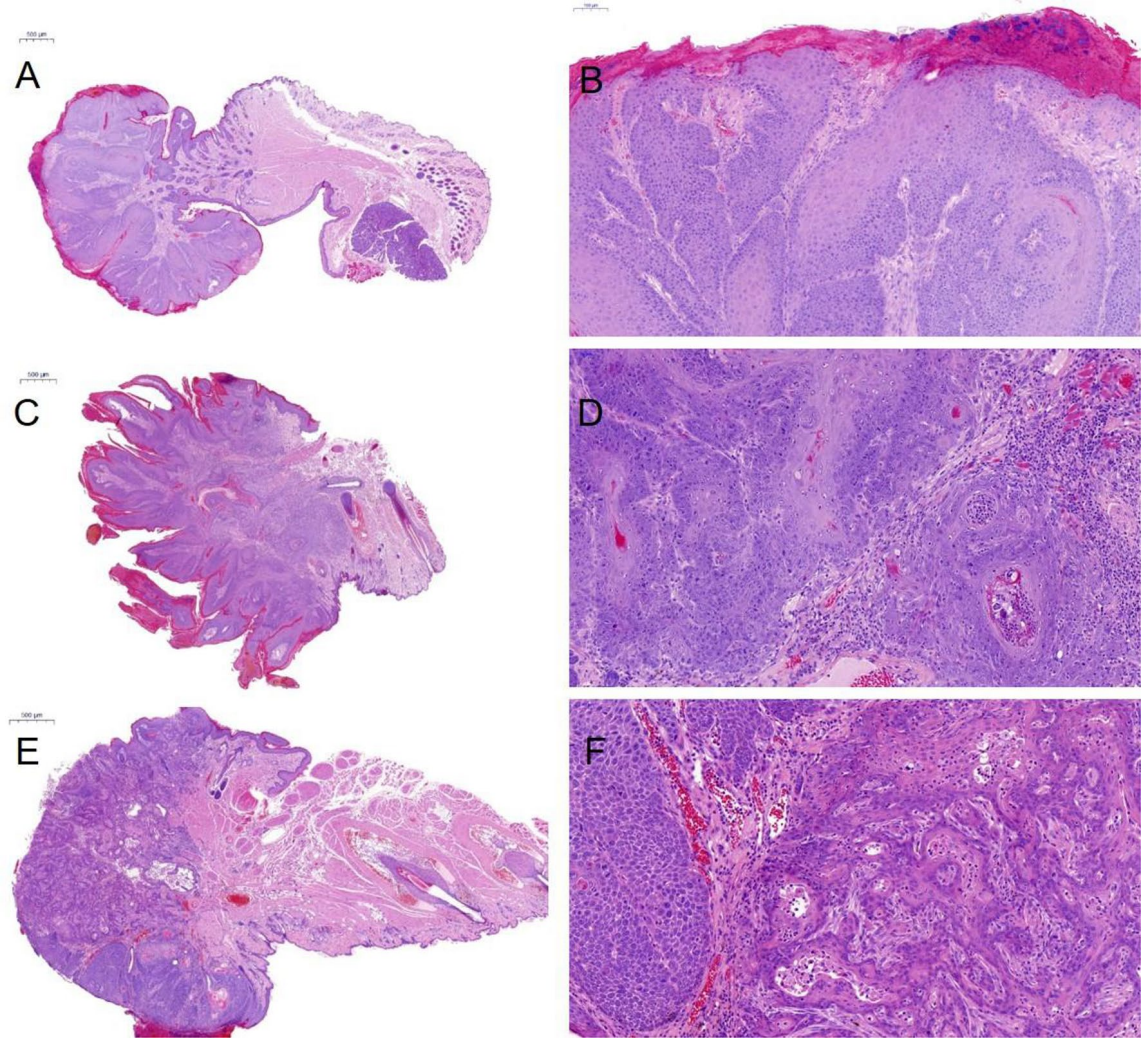
Representative histological analysis of squamous cell carcinoma (SCC) formation in the mucosa. Haematoxylin–eosin staining of squamous cell carcinoma (SCC) of the oral mucosa of mice [(**A**, **B**): MWM + high dose dibenzo(a)pyrene (DBP); (**C**, **D**) ultraviolet (UV) 48 s + high dose DBP; (**E**, **F**): kINPen 10 s + high dose DBP]. SCCs showed an endophytic and plump invasive growth pattern with mostly mild to moderate atypia.

**Figure 5:**
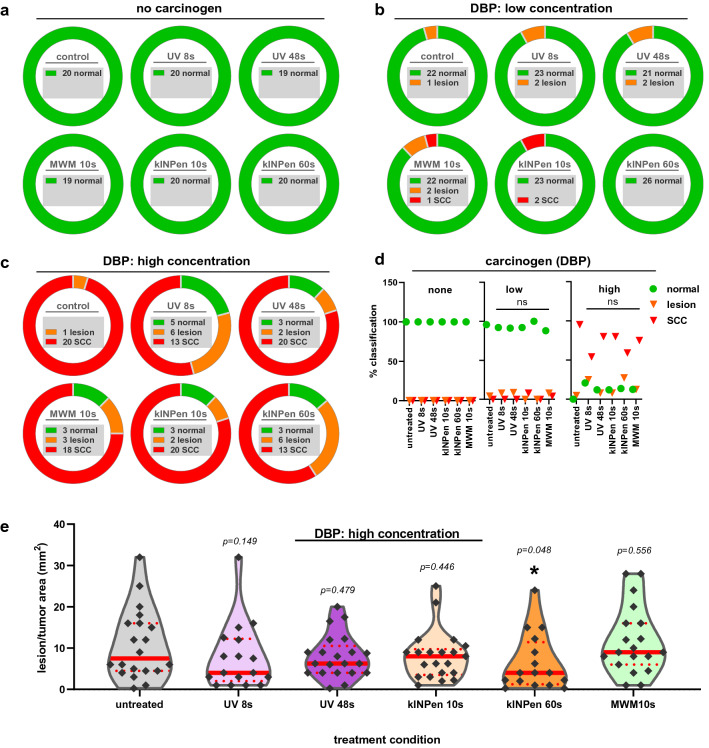
Frequencies and size of lesions and squamous cell carcinoma (SCCs). (**a**) the number of lesion-free and animals across all groups without administration of the co-carcinogen dibenzo(a)pyrene (DBP) indicating an absence of lesions with either of the treatments; (**b**) the number of lesion-free and animals across all groups with low dose (8 nmol) administration of the co-carcinogen DBP indicating the occurrence of 1–2 lesions or SSCs with either of the treatments; (**c**) the number of lesion-free and animals across all groups with high dose (24 nmol) administration of the co-carcinogen DBP indicating the occurrence of less than 20% of lesions or SSC-free animals with either of the treatments; (**d**) summary of results shown as percent histological result per DBP administration and treatment subgroup; (**e**) violin plots with single data showing the median (full red line) and 25% percentile (dotted red line) suggesting a lesion and SCC-reducing effect of 60 s of kINPen plasma treatment. Statistical analysis was performed using Fisher exact tests (**d**) and Kruskal–Wallis one-way analysis of variances (**e**) with *p* < 0.05 considered to be statistically significant (*); n = 19–26.

Mucosa tissue was investigated based on histopathological analysis. Non-invasive lesions were classified as: (1) hyperkeratosis (increased keratinization; Fig. 3A, B), (2) hyperplasia (expansion of the epithelial layer; Fig. 3C, D), (3) mild to moderate dysplastic changes (Fig. 3E, F), (4) papilloma without dysplasia (Fig. 3G, H), and (5) papilloma with moderate dysplastic changes (Fig. 3I, J). SCC invasive tumors showed a squamous differentiation with a partly exophytic growth pattern and plump invasion into the surrounding soft tissue (Fig. 4). Based on this classification, all mice in the DBP none-group lacked any histopathological signs of non-invasive lesions or SCCs in the oral cavity (Fig. 5a), irrespective of CAP treatment duration or the CAP device used. The same occurred for UV treatment. Low DBP doses caused a maximum of 10% histopathological alterations across all groups of either preneoplastic or SCC origin (Fig. 5b). Differences in the risk profiles were subtle, and CAP application did not generate excess precancer or cancer mortality (data not shown). High DBP doses caused either non-invasive lesion (20–25%) or SCC (55–95%) in the majority of mice irrespective of CAP exposure (Fig. 5c). Accordingly, frequency distributions for the occurrence of non-invasive or SCC lesions did not differ significantly between mice treated with either kINPen plasma, MWM plasma, or UV from mice of the untreated control group when separately analyzed for each DBP dose (Fig. 5d).

Interestingly, mice challenged with high dose DBP and frequently exposed to the kINPen plasma for 60 s showed a significantly reduced lesion/SCC area compared against untreated mice of the same DBP regimen (Fig. 5e). These data suggested the repeated plasma treatment was not harmful to the oral mucosa as no increase of lesions and SSCs was observed in any DBP regimen (no DBP, low DBP, high DBP). While plasma treatment did not prevent non-invasive lesion or SCC formation, it reduced their size for repeated 60 s kINPen exposure, indicating a potentially beneficial effect.

### CAP treatment of mucosa elicited minor changes in gene expression profiles

**Figure 6:**
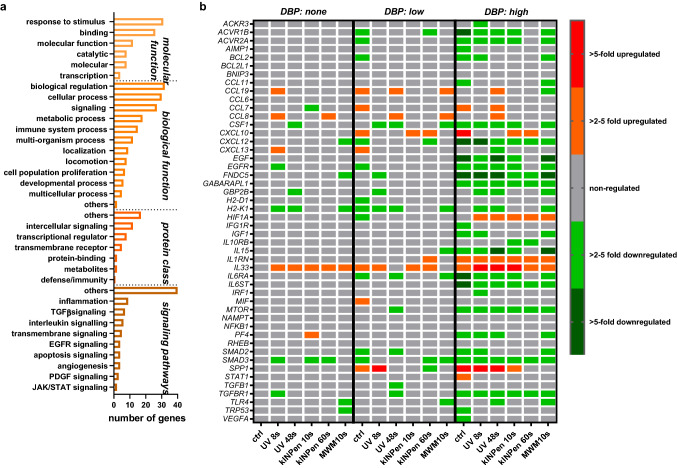
Gene expression analysis of 49 transcripts expressed and identified across all groups. (**a**) classification of identified genes across sorted by their occurrence within functions, pathways, and classes; (**b**) heatmap of relative gene expression for all targets and groups. Data are mean from duplicates of RNA pooled from the mucosa of 5 mice per group and normalized to the house-keeper *GAPDH* before the second normalization of untreated control mice's respective values.

**Figure 7:**
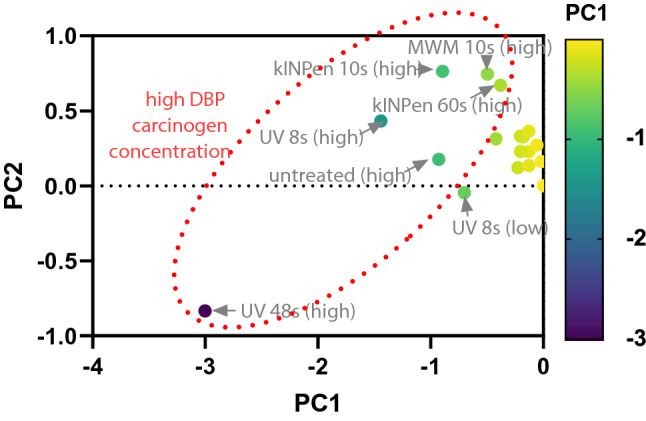
Principal component analysis of gene expression. Only genes consistently expressed across all groups were included for each treatment group and regimen in the principal component analysis (PCA), indicating the dibenzo(a)pyrene (DBP) high regimen (arrowheads) to differ in general much greater from the DBP low and the DBP none groups, in which the plasma or ultraviolet treatment only gave a minor effect (bubble cluster of all samples at the right side).

Subsequently, we performed gene expression analysis 1 year after CAP or UV treatment to define mucosal tissue's specific transcriptomic changes. From 144 targets of tumor formation and progression (Table 1), 49 genes showed differential regulation across the 17 groups within the three DBP regimens (no DBP, low DBP, high DBP) than untreated control tissue of mice not receiving DBP. Next, we used functional gene ontology classification to classify transcripts putatively involved in *molecular functions*, *protein classes, biological processes,* and *signaling pathways* (Fig. 6a). Among the main categories, we identified several classes such as *binding*, *intercellular signaling*, *transcriptional regulators*, and *response to stimul*i, which accounted for a significant contribution. For tumor formation and progression, several signaling pathways were found, including genes related to epidermal growth factor receptor (EGFR), transforming growth factor-beta 1 (TGFβ), interleukin, the Janus kinase/signal transducers and activators of transcription (JAK/Stat), apoptotic, angiogenetic, and inflammatory signaling pathways. When analyzing the fold-changes of expression across the 49 genes, neither the plasma nor the UV treatments provoked a substantial expression profile change in the animals not receiving any DBP (Fig. 6b). Except for *IL33*, we did not observe consistent or dose-dependent changes for any plasma or UV treatment. In principle, a similar finding was detected within the DBP low dose regimen, showing a slightly higher number of overall changes, while most changes occurred in the DBP high dose regimen, with a notable increase of *HIF1A* and *IL1RN* with most treatments. Across all regimens and treatments, fairly consistent changes occurred for *IL33*, *H2-K1*, and *SMAD3*. Changes in transcript expression levels of the chemokines *CCL19*, *CCL8*, and *CXCL12* were also frequent but instead found in the non-kINPen conditions. The more pronounced signature of changes in the high dose DBP regimens was also reflected in all groups' principal component analysis (PCA) (Fig. 7). All treatments within this regimen differed highly in PC1 and/or PC2, with the UV 48 s treatment differing the most. By contrast, except for UV 8 s (low dose DBP), all other samples clustered together. This result suggests the overall effect of the plasma or UV treatments on the gene expression profiles in the low dose and none DBP-regimens to be minor compared to the profound changes observed with high dose DBP per se.

**Table 1:** List of the 144 studied genes sub-grouped to different categories.

Groups	Gene symbol
**Immunity and inflammation**	
Immuno-stimulation	*IFNG, IL2, IL12A, IL12B, IL15, TNF*
Immuno-suppression	*PDL1, CSF2, CTLA4, CXCL12, CXCL5, IDO1, IL10, IL13, IL4, IL5, MIF, NOS2, PDCD1, PTGS2, TGFB1, VEGFA*
Enzymatic modulators	*AICDA, GZMA, GZMB, IDO1, NOS2, PTGS2*
Antigen presentation	*H2-D1, H2-K1*
Chemokines, cytokines, growth factors, and their receptors	*CCL2, CCL4, CCL5, CCL20, CCL22, CCL28, CXCL1, CXCL2, CXCL5, CXCL9, CXCL10, CXCL11, CXCL12, CCL24, CCL3, CCL7, CCL1, CCL11, CCL12, CCL17, CCL19, CCL6, CCL8, CCL9, CX3CL1, CXCL15, CXCL4, IL1A, IL1B, IL2, IL4, IL5, IL6, IL10, IL12A, IL12B, IL13, IL15, IL17A, IL22, IL23A, IL11, IL16, IL17B, IL17F, IL21, IL27, IL3, IL33, IL7, IL1RN, KITL, MIF, SPP1, TNF, TNFSF10, AIMP1, NAMPT, OSM, CSF1, CSF2, CSF3, EGF, EGFR, IGF1, TGFB1, VEGFA, CXCR7, CCR1, CCR2, CCR4, CCR5, CCR7, CCR9, CCR10, CXCR1, CXCR2, CXCR3, CXCR4, CXCR5, IL1R1, CCR3, CCR6, CCR8, IL10RA, IL10RB, IL5RA, IL6RA, IL2RB, IL2RG, IL6ST*
**Signal transduction**	
Interferon-responsive genes	*CCL2, CCL5, CXCL9, CXCL10, GBP2B, IRG1, MYD88, STAT1, TLR3, TNFSF10*
NFκB targets	*BCL2L1, CCL2, CCL5, CSF1, CSF2, CSF3, IFNG, TNF*
STAT targets	*CCL2, CCL4, CCL5, CSF1, CSF2, CSF3, CXCL9, CXCL10, CXCL11, CXCL12*
Toll-like receptor (TLR) signaling	*MYD88, TLR2, TLR3, TLR4, TLR7, TLR9*
**Apoptosis**	
Pro-apoptotic	*FASL, TNF, TNFSF10, TRP53*
Anti-apoptotic	*BCL2, BCL2L1, MYC, STAT3*
**Tumor necrosis factor and ligand**	*LTA, LTB, CD40LG, TNFSF11, TNFSF13, TNFSF13B, TNFSF4, TNFRSF11B*
**Myokines**	*ACVR1B, ACVR2A, AIFM1, BMP2, BNIP3, FNDC5, FST, GABARAPL1, IGF1R, MTOR, RHEB, SMAD2, SMAD3, TGFBR1*
**Transcription factors**	*FOXP3, HIF1A, IRF1, MYC, NFκB1, STAT1, STAT3, TRP53*

Interestingly, the tumor promoter transcript of osteopontin (*SPP1*) was highly upregulated in the control and UV treatments with high dose DBP, while plasma treatment strongly attenuated its expression. Moreover, the 60 s kINPen plasma treatment, which reduced absolute lesion and tumor sizes significantly, was the only treatment not showing a reduction in *ACVR1B*, *ACVR2A*, *CSF1*, *EGF*, *EGFR*, *IL15*, and *IL6RA* when compared to all other treatments in the high dose DBP group. In summary, our data suggest that repeated plasma exposure be void of carcinogenic effects when applied alone or in combination with the co-carcinogen DBP. Also, the overall changes in gene expression in the plasma treatment groups were minor in the DBP none and low dose regimens, while being somewhat more pronounced in the DBP high dose regimen, which might be a consequence of the tumor-size-reducing properties observed mainly with the 60 s kINPen treatment.

## Discussion

Diseases in the oral cavity such as peri-implantitis need improved treatment options. Preclinical data suggest cold atmospheric pressure plasma (CAP) as a promising tool to target peri-implantitis and promote re-osseointegration. Novel therapeutic avenues in medicine, however, require both efficacy and safety. To this end, in the present proposal, we assessed the safety and carcinogenic potential of two market-ready CAP devices after repeated CAP exposure of the oral mucosa over 1 year in a vast mouse collection. CAP treatment neither provoked preneoplastic lesions or tumor formation nor enhanced such mucosal alterations in mice repeatedly challenged with the co-carcinogenic substance DBP, which was administered in two different concentrations in analogy to Guttenplan et al.^12^: 3 nmol (low dose) and 24 nmol (high dose). The low amount of DBP was chosen to detect a subthreshold cocarcinogenic effect possibly. The tumor development in these groups is similar to the results already described^12^.

We recently reported a single 60 s kINPen plasma treatment to cause superficial ulceration and inflammation compared to 10 s treatment after 1 day in mice^10^. Nevertheless, the repeated CAP treatment did not exert permanent mucosal damage during this 1-year study. The repeated-exposure study design simulates the clinical situation, e.g., putative CAP maintenance sessions of one or up to six implants every 3 to 6 months. Hence, disease management often requires multiple CAP treatment cycles, but CAP's long-term carcinogenic risk profile in the oral cavity is unknown. Our data provide evidence that CAP exposure with two different plasma devices and different treatment times monthly repeated over 1 year is not carcinogenic in mice, at least under our experimental setting. This finding is in line with our previous report suggesting the safety of repeated exposure of sterile wounds to the kINPen plasma^13^. None of the healed wound tissues showed signs of preneoplastic or neoplastic lesions 1 year after several treatment cycles. Similar findings come from a range of internal organs and tumor-associated markers in the blood plasma, supported by NMR and PET-CT imaging. At the same time, wound healing was promoted in a similar setting^14^. The notion of safe CAP application also derives from a 1-year follow-up study in human wounds^15^, previously exposed to the kINPen plasma, to demonstrate its wound healing-promoting properties^16^. These findings were recently confirmed in the same cohort using in vivo confocal laser scanning microscopy and hyperspectral imaging, revealing a lack of malignant changes, inflammatory reactions, or pathological modifications in the tissue exposed to the plasma treatment 5 years earlier^17^. Moreover, a meta-analysis based on 9 RCTs (268 patients) concluded that CAP is safe in wound care^18^. However, the safety of CAP treatment should be monitored closely in humans, especially in the cases of repeated use and/or prolonged treatment time. Observational studies in humans should also be conducted for this purpose in the future. Furthermore, additional preclinical investigations with different protocols to the one used in the present study would be highly beneficial to substantiate or disprove the safety of CAP treatment.

UV radiation is known to cause cellular damage that can ultimately lead to cancer. On the one hand, UV radiation has direct effects on cellular molecules due to energy absorption (DNA damage). On the other hand, UV radiation induces the release of intracellular reactive species^19^. UV radiation is also well-described for CAP, which is well below potentially dangerous intensities for the kINPen^14^. Indeed, the helium/nitrogen-operated MWM jet generates only about 10% of the effective UV irradiation of the kINPen^20^. Repeated exposure with neither of the jets favored cancer development in our model. Since both UV control groups (8 s and 48 s) were exactly intensity-matched to the kINPen plasma treatment UV generation and both did not show any difference from the control or CAP groups, we are confident that the UV component of CAP neither causes damage nor promotes carcinogenesis.

Another well-known CAP product is ROS/RNS. As these frequently occur during chronic inflammation, a driver of cancerogenesis^21^, ROS/RNS formation often is falsely set equal to be tumor-promoting. However, ROS/RNS biology is more complex, as these molecules play a pivotal role in several redox relays and signaling pathways, maintaining physiological processes^22^. ROS/RNS are also indispensable for pathogen defense^23^. Notably, the specific locus of ROS/RNS formation is critically decisive to the consequence of their presence. Intracellular ROS/RNS, generated, for instance, during intense UV radiation and radiotherapy close to the DNA in the form of hydroxyl radicals, are established DNA damaging agents and promote mutagenesis^24^. CAP, however, generates extracellular ROS/RNS^8^. Although these ROS/RNS may induce lipid peroxidation, and some of them may enter the intracellular compartment, their high reactivity with abundant cellular thiols and membranes along with low diffusion distances prohibits any entering of the nucleus per se. Accordingly, we previously provided evidence that the phosphorylation of the histone A2X, a known marker of DNA double-strand breaks in radiobiology, is a consequence of apoptotic processes rather than the direct action of CAP-derived ROS/RNS on the DNA^25^. Along those lines, human oral mucosa tissue exposed to the kINPen plasma for up to 300 s ex vivo did not show any H2AX phosphorylation^26^. Other evidence supports the lack of genotoxicity of CAP treatment. For the kINPen plasma device, the OECD-accredited HRPT-assay^27^ as well the cytokinesis-block micronucleus assay in vitro^28^ and in ovo^29^ did not identify increased frequencies across a range of conditions. Similar results came from experiments using other plasma sources^30–32^.

Our qPCR screening revealed a lack of CAP-specific changes across all DBP regimens (no DBP, low DBP, high DBP). In summary, genes' distribution clearly shows that the carcinogenic substance DBP dose is critical. The most consistently increased target in both CAP and non-CAP treatment was IL33, which is upregulated in oral lichen planus^33^ and periodontal disease^34^. Oral lichen planus^35^ and SSC^36^ are associated with elevated SMAD3 levels, a target that we found frequently decreased and links into TGFβ-signaling (*TGFB1*, *TGFBR1*)^37^. *SPP1* codes for osteopontin, which is elevated in SCC^38^.

Interestingly, 60 s kINPen and 10 s MWM plasma treatment decreased its marked upregulation observed in the other high dose DBP groups. Another notable finding was the increase of *HIF1A* in all but the control group in this regimen. This transcription factor is constitutively increased in SCC^39^, the decreased lesion/SCC size with the 60 s kINPen plasma treatment, and the lack of increased frequencies of preneoplastic lesions or SCCs with any of the treatment suggest its increased transcription not to link to tumor progression. Among low- and high-dose DBP regimens, kINPen plasma exposure increased *CXCL10* levels. CXCL10 is a double-edged sword related to inadequate radio-therapeutic response in SCC on the one hand^40^ and promotion of antitumor-immunity and SCC decline on the other^41^. We previously linked CAP-mediated antitumor-immunity to increased CXCL10 release^42^ and identified CAP treatment to decelerate UV-induced preneoplastic lesion development in vivo^43^, underlining the promising finding of CAP-reduced lesion/SCC sizes in the current work that warrants further exploration in the future.

## Conclusion

One year of repeatedly applied CAP treatment of the oral mucosa was well tolerated, and no carcinogenesis occurred. This finding was independent of the CAP device (kINPen vs. MWM) and treatment time investigated. As the UV controls did not show any detrimental effect, we can conclude that both plasma jets' low UV emission is safe when repeatedly applied to oral tissue. Moreover, CAP treatment of the oral mucosa challenged with the co-carcinogen DBP did not promote its harmful effects, as seen with a similar or lower number of animals presenting with preneoplastic or SCC lesions. The significantly reduced size of the preneoplastic lesions or SCC in mice receiving high-dose DBP and 60 s of kINPen plasma treatment even suggests a benefit of the CAP exposure against (pre)malignant development. These findings agree with previous reports on the safe application of these CAP devices and motivate their future use in patients to promote implants' re-osseointegration and improve oral implantology health care.

## Materials and methods

### Animals

Six to eight-week-old male mice (n = 455, B6C3F1, Charles River Laboratories, Sulzfeld, Germany), weighing 25–30 g, were housed under standard conditions in Makrolon type III cages. There was a 12/12-h light/dark rhythm and access to water and pelletized food ad libitum. The conditions of the animals were monitored several times a day. The study was approved by the Committee for Animal Research (Landesamt für Landwirtschaft, Lebensmittelsicherheit und Fischerei, Rostock, LALLF; approval number AZ 7221.3-1-057/13) following the German Animal Protection Law. All experiments performed and reported here were following the ARRIVE guidelines. Out of 455 mice, 49 animals were excluded for several reasons (e.g., death during narcosis at the beginning of the experiments), leaving 406 mice for analysis. One hundred ten animals had been killed preterm because of large tumors. A total of 19–26 animals were finally allocated to each of the 18 groups (Fig. 1).

### Study design

A 3 × 6 study design was chosen. Due to a high prevalence of smokers in the population, tobacco's co-carcinogenic effect was investigated by administering either no DBP, low dose (3 nmol), or high dose (24 nmol) DBP. DBP was diluted in DMSO^12^ and administered into the oral cavity three times per week in two different concentrations (3 nmol = 'low'; 24 nmol = 'high') for the entire study period of 12 months.

While high doses are known to promote tumor formation, low doses of the co-carcinogen together with other putative carcinogenic inducers potentiate lesion formation. Within these three DBP groups, six treatment modalities were applied.

Two plasma sources were investigated. The first was the atmospheric pressure argon plasma jet kINPen 09^44,45^. The kINPen uses argon (Air Liquide, Düsseldorf, Germany) as working gas with a flow rate of 3 standard liters per minute (sL/m), and treatment times were 10 s and 60 s. The second plasma source was a microwave pulsed plasma jet (MWM, Leibniz Institute of Surface Engineering, Leipzig, Germany). It was operated with helium and nitrogen (Air Liquide, Düsseldorf, Germany) as feed gas with a flow rate of 3 sL/m as described before^20^ and a 10 s treatment time. The distance between the tip of the plasma effluent and the mucosa was between 7 and 10 mm. Animals without any treatment served as a negative control. A UV source (Xe flashlight with a power supply; Voltcraft Conrad Electronic, Wollerau, Switzerland) with radiation energy of 155 μW/cm^2^ and a UV spectrum comparable to that of the kINPen 09 (UVA/B 119 μW/cm^2^ ^46^) was used to assess possible carcinogenic effects of UV emission. Exposure times (8 s and 48 s) were radiation-matched to the kINPen treatment times (10 s and 60 s). Mice received weight-adjusted anesthesia consisting of 50 mg/kg ketamine and 10 mg/kg body weight xylazine (both Selectavet Dr. Otto Fischer, Weyarn, Germany) intramuscular injection before CAP and UV treatment. The right cheek mucosa was kept open with anatomical plastic forceps (Mediware Servoprax, Wesel, Germany) and continuously treated with CAP or UV. The 60 s CAP treatment period was divided into six intervals of 10 s with 10 s breaks between the treatments. Treatment was performed once per month over 1 year. The contralateral left side was left untreated. Additionally to the five treatment subgroups, one subgroup was left untreated.

Animal weight was monitored monthly before treatment and before sacrifice. Mice with high tumor burden were sacrificed before that endpoint according to predefined score criteria. One month after the last treatment cycle, all animals were sacrificed by cervical dislocation, followed by complete autopsy and cheek tissue analysis.

### Tissue analysis

After sacrifice, the oral mucosa was examined macroscopically, and the cheeks were explanted. After fixation in buffered formalin for 24 h, the samples were dehydrated by standard techniques and embedded in paraffin. Afterward, 4 µm thick sections were cut with a microtome and stained with hematoxylin and eosin (H&E) and the Periodic Acid-Schiff (PAS) reagents. Two investigators analyzed the sections microscopically (Nikon eclipse ci-L; Nikon, Tokyo, Japan) independently.

### Cheek mucosa sample preparation, RNA isolation, and quantitative PCR

Mucosa tissue was stored in liquid nitrogen until homogenized using a gentle MACS Dissociator (Miltenyi Biotec, Bergisch Gladbach, Germany) in Qiazol buffer. According to the manufacturer's instructions, RNA was isolated using the *RNeasy Plus Universal Midi Kit* (Qiagen, Hilden, Germany). The tissue of five mice per group was pooled, and one µg of pooled RNA was used for cDNA synthesis with the first-strand cDNA synthesis kit (Qiagen, Hilden, Germany). Quantitative (q)PCR was performed using four customized panels (custom RT^2^ profiler PCR array; Qiagen, Hilden, Germany) with gene-specific primers and RT^2^ SybrGreen qPCR mix in two technical replicates in a thermocycler (7900HT Fast Real-Time PCR System; Applied Biosystems, Waltham, MA, USA). One hundred forty-four genes belonging to different subgroups were analyzed (Table 1). The data analysis was based on the _ΔΔ_CT method, in which the raw data were normalized to the housekeeping gene *GAPDH*. Differential gene expression of the treated mice was normalized to untreated control. Analysis of the genes' function was conducted using the Protein Analysis Through Evolutionary Relationships (PANTHER) classification system and gene ontology (GO) pathway analysis (open database).

### Sample size derivation

The number of mice per group was planned based on sample sizes from a previous study (Guttenplan)^12^ and following the local animal experiment authority. Because sample sizes of 20 mice (real numbers of mice were between 16 and 20) were sufficient to demonstrate that frequencies of oral mucosal abnormalities differed significantly according to DBP concentrations^12^, we initially chose sample sizes such that numbers of mice were at least 20 for each group. Thus, we increased the number of mice within each group up to 31 mice, finally achieving effective sample sizes between 19 and 26 in this study.

### Statistical analyses

In descriptive analyses, differences in frequencies and percentages of histologic mucosa alterations (normal versus non-invasive lesions versus SCCs) for all treatments (UV8 s, UV48 s, kINPen plasma 10 s, kINPen plasma 60 s, and MWM plasma 10 s) compared to untreated controls within groups defined by DBP dose (low DBP dose, and high DBP dose; tests were omitted for "no DBP" due to missing distributional variation) were examined with Fisher exact tests; *p* values were corrected for multiple testing using the Bonferroni-Hochberg procedure. For comparison of lesion and SCC sizes, the Kruskal–Wallis test was used, and *p* ≤ 0.05 (*) was considered significantly different.

For each group (no DBP, low DBP, high DBP), weight change was modeled using mixed-effects models with random intercepts and random slopes for a time across mice (Fig. 2). Fixed effects included treatment group and time (in same months; modeled as restricted cubic splines with three knots, thereby allowing for non-linearity). By including the interaction term between group and time, group-dependent differences in weight rates over time were identified. For graphical presentation, predicted means were calculated using fixed terms only.

Two-sided *p*-values ≤ 0.05 were considered statistically significant. Analyses were performed using the Stata/SE version 14.2 (StataCorp LP, College Station, TX, USA).

For principal component analysis, the center method was used to select PCs with eigenvalues greater than 1.0. *Prism* 9.0 (GraphPad Software, San Diego, CA, USA) was used for statistical analysis and graphing.
